# The use of ketotifen as long-term anti-inflammatory prophylaxis in children with PFAPA syndrome

**DOI:** 10.3389/fimmu.2023.1302875

**Published:** 2023-12-07

**Authors:** Lenka Kapustova, Peter Banovcin, Anna Bobcakova, Eva Jurkova Malicherova, Daniela Kapustova, Otilia Petrovicova, Branislav Slenker, Adam Markocsy, Filip Oleksak, Karolina Vorcakova, Milos Jesenak

**Affiliations:** ^1^ Centre for Periodic Fever Syndromes, Department of Paediatrics, University Hospital in Martin, Jessenius Faculty of Medicine of Comenius University in Bratislava, Martin, Slovakia; ^2^ Department of Pneumology and Phthisiology, University Hospital in Martin, Jessenius Faculty of Medicine of Comenius University in Bratislava, Martin, Slovakia; ^3^ Clinic of Dermatovenerology, University Hospital in Martin, Jessenius Faculty of Medicine of Comenius University in Bratislava, Martin, Slovakia; ^4^ Department of Clinical Immunology and Allergology, University Hospital in Martin, Martin, Slovakia

**Keywords:** PFAPA syndrome, children, flares, ketotifen, prophylaxis

## Abstract

**Introduction:**

Periodic fever, aphthous stomatitis, pharyngitis and adenitis syndrome (PFAPA) is the most frequent periodic fever syndrome in children. Its pathogenesis is still unknown, but some disease-modifying factors were observed. Several medications were tested for the long-term prophylaxis of inflammatory flares; however, none are standardly used.

**Methods:**

This prospective clinical trial enrolled 142 children (71 girls, 50%) meeting diagnostic criteria for PFAPA syndrome. We analysed selected clinical characteristics and compared laboratory parameters during the flare and attack-free period (at least two weeks after the attack). Moreover, we assessed the possible therapeutic effect of ketotifen on the duration of attack free-periods and clinical picture.

**Results:**

The mean age of patients was 6.81 ± 3.03 years and the mean age of onset of symptoms was 2.31 ± 2.02 years. No significant differences were observed between genders.We recorded a positive family history for PFAPA in 31.69% of patients. Attacks lasted for 2.8 ± 1.2 days, with intervals between attacks of 4 ± 1 weeks. We administered ketotifen in 111 (77.8%) patients, and a positive effect was observed in 86 (77.5%) of patients. We observed prolonged attack-free intervals in patients treated with ketotifen (14.7 ± 8.9 days in comparison with 4.4 ± 1.9 days before the treatment; p<0.001). The used dose of ketotifen was 0.08 ± 0.01 mg/kg/day. Mild side effects were observed in four patients (restlessness, irritability, agitation and constipation).

**Discussion:**

Our data supports the use of ketotifen for long-term prophylaxis in children with PFAPA syndrome with positive effects on the attenuation of disease activity and the prolongation of attack-free periods. Further well-designed studies should confirm the preliminary data.

## Introduction

Periodic fever, aphthous stomatitis, pharyngitis and cervical adenitis syndrome (PFAPA) is the most common periodic fever syndrome in childhood. In the terms of aetiology, it is considered to be an idiopathic disease. A polygenic pattern of inheritance is suspected, probably in combination with some epigenetic factors. The incidence is 2.3 per 10,000 children below the fifth year of life (1) with a mild predominance of boys. However, it seems that this disease is underdiagnosed in general and seems to be more frequent in this age group. The first description of this clinical entity comes from the case series published by Marshall et al. in 1987 (2). The clinical criteria, which are still used in clinical practice with slight variations, come from Thomas et al. from 1999 (3). In the clinical criteria according to this group, we include: 1) regularly recurring attacks of fever (usually more than 39°C, without reaction to common antipyretics) with an onset before the 5^th^ year of life; 2) at least one of the symptoms: aphthous stomatitis, pharyngitis and cervical lymphadenopathy; 3) complete asymptomatic intervals between attacks (so-called flares); 4) the exclusion of cyclic neutropenia; and 5) normal growth and development of the child. Clinical symptomatology may also include some minor symptoms such as abdominal pain, arthralgia, headache, myalgia, nausea, vomiting and skin manifestations (4, 5). Some patients also experience prodromes a few days before the attack onset (6). The duration of attacks is usually 3 to 7 days, most often 4 to 5 days, with an interval between individual attacks of 2 to 8 weeks, which is 3 to 6 weeks in most patients (3, 4, 7). The onset of the disease is assumed to be between the 4^th^ and 5^th^ year of life and it mainly affects boys (a more pronounced phenotype of the disease is assumed in boys). There are also studies demonstrating an earlier onset of disease (usually between 11 and 18 months of age) and also studies demonstrating later onset of disease (in adulthood) (1, 5, 8). Federici and Gattorno noted a significant cyclical pattern of episodes in children with PFAPA, even such that children’s parents were able to predict the attack and plan a vacation from work (9). However, this regularity can disappear after a certain period of time of the disease or after initiation of the episodic application of corticosteroids. The episodes gradually shorten and the intervals become longer until the disease spontaneously resolves, usually around the age of ten. The diagnosis of PFAPA syndrome is therefore based on clinical criteria and established *per exclusionem* after excluding other causes of recurrent fever. In new inclusion diagnostic criteria produced by the CARRA (Childhood Arthritis and Rheumatology Research Alliance) PFAPA working group, a positive therapeutic response to corticosteroids was also included in the diagnostic criteria (10). Positive therapeutic response means termination of the attack at the maximum dose of prednisone (2 mg/kg/dose) in one or two doses within 24 hours from the onset of fever.

PFAPA belongs to autoinflammatory diseases with probably oligogenic or polygenic heredity. The pathomechanism is still not known, but some of the pathogenic mutations in heterozygous state as well as variants of uncertain significance in the genes typically affected in familial Mediterranean fever (*MEFV* gene) and cryopyrinopathies (*NLRP3* gene) could also be present in PFAPA patients (11–14). The presence of these variants can affect the clinical picture and response to medication. Also in PFAPA the possible candidate gene was identified to be *SPAG7* (confirmed in only one patient in one study) (15). Di Gioa et al. did not confirm the significant importance of this gene in the pathogenesis of PFAPA. However, they found that each individual with PFAPA had more than one rare variant present in the inflammasome genes (16). Also, polymorphism in the *CARD8* gene (*CARD8-FS* variant, rs140826611) which can modify the clinical picture, was identified in patients with PFAPA (17). The probable genetic predisposition of the disease is also declared by the fact that approximately 23% of patients with PFAPA have one or more first-degree relatives with clinical symptomatology meeting the criteria for PFAPA. Parents of individuals with PFAPA have a significantly higher incidence of recurrent pharyngitis and recurrent aphthous stomatitis (18). In general, we can divide the therapeutic approach to a child with PFAPA into conservative and surgical treatment. Since febrile attacks usually do not respond to conventional antipyretic therapy, a single oral administration of corticosteroids (prednisone 1-2 mg/kg/dose or betamethasone 0.2 mg/kg/dose) is used to cease a febrile attack. The dose must be administered at the beginning of the attack and can be repeated on the second day if the effect of the first dose is insufficient or transient (6, 10, 19, 20). No significant difference in the effect of therapy was observed if the patients were given a lower dose of prednisone (1–2 mg/kg/dose vs. 0.5 mg/kg/dose) (20). However, corticosteroids may shorten the intervals between episodes of fever or disrupt the regularity of the attacks. The rapid resolution of symptoms after one or two doses of corticosteroids is also considered a positive therapeutic test typical for PFAPA (10).Different medications have been tried in the prophylaxis of frequent PFAPA attacks to date. Cimetidine (histamine H_2_ receptor antagonist) seemed to be ineffective for the resolution of frequent febrile episodes. Colchicine seems to be safe, well-tolerated, and effective in PFAPA patients. It can be considered in children with moderate or high disease activity even those without corticosteroid-benefit (21). Also, in the case of refractory aphthous stomatitis, colchicine was used successfully (22). The application of anakinra (recombinant receptor antagonist for interleukin-1) led to the rapid clinical and laboratory remission of a PFAPA attack (23, 24). In the literature, there is also a case report of an adult patient with PFAPA who was treated with canakinumab (a fully human monoclonal antibody against interleukin-1β) with good effect (25).

Ketotifen is the first-generation antihistamine with proven immunomodulatory effects. According to some studies it reduces the production of IL-1β and some proinflammatory chemokines and modulates the T cell response. The positive effect of ketotifen on reduction of frequent attacks was also demonstrated in a case series of PFAPA patients (26). Another interesting approach is the application of probiotics containing *Streptococcus salivarius K12*, which produces bactericidal substances that can interact with the growth of many bacteria and modulate immune response in oral cavity. Di Pierro et al. tried administering this oral probiotic in a form of slowly dissolving tablets. Three of the four patients had no PFAPA attacks during the 3 months of administration (27). The only surgical solution is tonsillectomy with/or without adenoidectomy. However, surgical therapy is reserved only for indicated patients who have frequent relapses of the disease lasting more than a year, without sufficient response to corticosteroids, and with a significant negative impact on the quality of life of the affected children. Some authors consider tonsillectomy to be the most effective therapy for PFAPA in children (19, 28).

In our trial, we aimed to analyse the selected clinical and laboratory characteristics of the children fulfilling the clinical criteria for PFAPA syndrome and assessed the possible preventive role of ketotifen on the onset of flares and disease activity.

## Material and methods

This prospective clinical study was conducted in the Centre for periodic fever syndromes in University Hospital in Martin, Slovakia. We enrolled the children who met clinical criteria for PFAPA according to Thomas et al. (3). Data were collected in the period of 2016-2019. Written informed consent was signed by the participants’ legal guardian/next of kin in all of the patients. The final group of patients consisted of 142 children (71 girls and 71 boys). All of the patients were asked to come for regular check-ups (usually in the interval of 3-6 months from the first examination). We characterised the clinical picture in all of the patients and performed blood sampling for selected laboratory inflammatory markers during the attack and attack-free periods (sampling performed at least two weeks after the attack). In selected markers we included complete blood count with a differential leucogram, C-reactive protein (CRP), procalcitonin (PCT), interleukin-6 (IL-6), alpha 1-acid glycoprotein (AGP), immunoglobulin D (IgD), serum calprotectin (sCAL), serum amyloid A (SAA) and vitamin D. In the second part of the study, we focused on the possible treatment options with the main focus on therapeutic effect of ketotifen as a long-term prophylaxis of frequent attacks, dose needed and safety and possible side effects of this treatment. Attack flares were reported by parent and evaluated by physician in our Centre for periodic fever syndromes. Each PFAPA patient has a disease activity diary, which we evaluated with a parent to clarify it was a PFAPA attack. We used standard dose of ketotifen recommended for children: children older than 3 years of age—the usual dose is 1 milligram (1 tablet) twice daily, once in the morning and once in the evening; infants and children from 6 months to 3 years of age—dose based on body weight (0.05 mg/per kilogram of body weight) twice daily, once in the morning and once in the evening. We evaluated the frequency of attacks before the treatment with ketotifen and at least 3-6 months from the beginning of the treatment. For this study we considered doubling the interval between the episodes as the positive prophylactic effect.

All the results were statistically processed using *MedCalc 11.5.1.0.* (*MedCalc Software bvba, Ostend, Belgium).* We performed summary statistics for all parameters with determination of the distribution of parameters using the Shapiro-Wilk test. Quantitative data with a parametric distribution were expressed as the average (avg.)± standard deviation (SD). For non-parametric data we determined the median and interquartile range (IR; between the 25^th^ and 75^th^ percentiles). We used the Mann-Whitney non-parametric test or independent t-test to analyse and determine the significance level between groups with non-parametric distribution. For data with a parametric distribution, we used Student’s t-test. We evaluated the qualitative data using the Chi-square test, in groups with the number of individuals less than ≤ 5 we used Fisher’s test. We expressed the qualitative data as the number of individuals (N) and in the percentage (%). The level of significance was expressed using the p-value. We considered statistically significant values ​​p<0.05(*), more significant p=0.05–0.001(**); and most significant p<0.001(***).

## Results

In our Centre for periodic fever syndromes, we first identified 156 patients meeting the criteria for PFAPA, but 14 were excluded (12 because they did not come to regular check-up and in 2 of them, we have confirmed familial Mediterranean fever, both clinically and genetically). The final group of patients consisted of 142 children (71 girls and 71 boys). Both genders were represented equally (50%). The characteristics of the patients are included in **Table 1**.

**Table 1:** Demographic characteristics of the studied cohort.

Number of patients (N, %)	Whole group 142 (100%)	Boys 71 (50%)	Girls 71 (50%)	p-value
Age (in 01/2019) (years, avg.±SD)	6.81±3.03	6.79±2.85	6.83±3.21	0.94
Age of first symptoms (years, avg.±SD)	2.31±2.02	2.46±1.90	2.16±2.14	0.27
First symptoms<1^st^ year of life (N, %)	21 (14.8%)	10 (7.04%)	11 (7.76%)	0.81
First symptoms≥1^st^ <3^rd^ year of life (N, %)	74 (52.11%)	40 (28.17%)	34 (23.94%)	0.31
First symptoms≥3^rd^ year of life (N, %)	47 (33.1%)	21 (14.79%)	26 (18.31%)	0.37
Age of diagnosis of PFAPA (years, avg.±SD)	4.17±2.72	4.31±2.78	4.03±2.67	0.52
Diagnostic delay (years, avg.±SD)	1.86±0.70	1.85±0.88	1.87±0.53	0.83

N, number of patients; avg., average; SD, standard deviation. We calculated the percentage representation in this table from the entire number of patients (N=142). The p-value is calculated between genders.

The average age of patients in our group (counted for 01/2019) was 6.81 ± 3.03 years, we did not notice any significant differences by gender. The age of the first symptoms was 2.31 ± 2.02 years. In the group of boys compared to girls, this age was slightly higher (2.46 ± 1.90 vs. 2.16 ± 2.14 years), but not statistically significant (p=0.27). Interestingly, almost half of the patients had first symptoms between 1^st^ and 3^rd^ year of life. The age of diagnosis in our patients was 4.17 ± 2.72 years; for boys it was 4.31 ± 2.78 years and for girls was 4.03 ± 2.67 years (p=0.52). The diagnostic delay from the first symptoms to the establishment of the diagnosis was 1.86 ± 0.70 years (gender difference p=0.83). This can have a serious impact in terms of incorrect treatment of the patient and their quality of life.

### Family history and PFAPA

In our group, almost one third (31.69%) of patients had a positive family history for PFAPA (46.7% boys and 53.3% girls). Girls had a higher number of parents with clinical symptomatology of PFAPA than boys. Boys had a higher number of siblings with similar clinical symptomatology. A higher number of girls with PFAPA had more than two first-degree relatives with clinical symptomatology of PFAPA. However, differences between genders were not significant. We also recorded a positive family history of rheumatic diseases in 6 patients with PFAPA syndrome (4 patients with rheumatoid arthritis, 1 patient with Sjögren syndrome and 1 patient with Bechterev disease). Immunodeficiencies occurred in 4 relatives of patients with PFAPA - predominantly antibody immunodeficiencies (3 selective IgA deficiency) and the parent of one patient was carrier of a mutation for familial haemophagocytic lymphohistiocytosis.

### Characteristics of clinical symptoms

Attacks occurred in intervals of every 4 (3–5) weeks: in boys 4 (4–5) weeks, in girls 4 (3–5) weeks (p=0.2252). The mean duration of the attack was 2.8 ± 1.2 days (boys 2.8 ± 0.98 days, girls 2.8 ± 1.3 days; p=0.9823). Complete clinical expression (present of fever and all 3 pathognomonic symptoms) was present in 50.7% of patients (54.9% boys and 46.5% girls). Periodic fever was present in 100% of patients, pharyngitis in 94.4%, aphthous stomatitis in 54.9% of patients and cervical lymphadenopathy in 91.6% of patients. Among the non-pathognomic symptoms, we noticed arthralgias in 19.7% of patients, abdominal pain in 29.6%, skin rash in 7.8%, and accentuated fatigue at the time of the attack in 16.2% of patients. In one patient, we observed genital ulceration as a possible symptom of PFAPA. We found a significant difference in the higher occurrence of skin rashes during the attack in girls than in boys (12.7% vs. 2.8% respectively; p=0.0279). Other clinical symptoms did not show a significantly different distribution between genders (**Table 2**).

**Table 2:** Clinical presentation of PFAPA syndrome in studied patients.

	Whole group N=142	Boys N=71	Girls N=71	p-value
Attack duration (days)	2.8±1.2	2.8±0.98	2.8±1.3	0.9823
Attack-free interval (weeks)	4 (3–5)	4 (4–5)	4 (3–5)	0.2252
Fever	142 (100%)	71 (100%)	71 (100%)	NS
Pharyngitis	134 (94.4%)	67 (85.9%)	67 (85.9%)	NS
Aphthous stomatitis	78 (54.9%)	43 (60%)	35 (49.3%)	0.1773
Genital ulcers	1 (0.7%)	0 (0%)	1 (1.4%)	NS
Cervical lymphadenopathy	130 (91.6%)	63 (88.7%)	67 (94.4%)	0.2275
Arthralgias	28 (19.7%)	11 (15.5%)	17 (23.9%)	0.2057
Abdominal pain	42 (29.6%)	17 (23.9%)	25 (35.2%)	0.1413
Skin rash	11 (7.8%)	2 (2.8%)	9 (12.7%)	0.0279
Fatigue	23 (16.2%)	10 (14.1%)	13 (18.3%)	0.4944
Complete PFAPA	72 (50.7%)	39 (54.9%)	33 (46.5%)	0.3139

NS, non-significant.Percentage is calculated for individual groups by gender and for the whole group. The p-value is calculated between genders. Individual values ​​are given as average± standard deviation for parametric variables and as median (interquartile range) for non-parametric variables.

### Characteristics of laboratory parameters

In the group of patients with PFAPA, we analysed selected laboratory parameters associated with inflammation. Comparing the data from the acute attack and attack-free period, we observed a significant elevation of CRP during the attack (50 (17.1–120) mg/L vs. 0.95 (0.3–2.3) mg/L, p<0.0001); SAA (132 (36.65–278.25) mg/L vs. 4 (3.4–7.43) mg/L, p<0.0001); IL-6 (6.94 (1.87–20.64) ng/L vs. 1.52 (1.5-2.89), p=0.0001); alpha-1 acid glycoprotein (1.51 (1.23–1.65) g/L vs. 0.94 (0.74–1.32) g/L, p=0.0013) and in some patients also elevation of procalcitonin (0.12 (0.07–0.55) µg/L vs. 0.06 (0.04–0.10) µg/L, p=0.0185). In the attack-free period we found a significant elevation of serum calprotectin (5.92 (3.11–12.8) µg/mL vs. 3.64 (2.94–6.28) µg/mL, p=0.036) as a possible marker of subclinical inflammation.

We noted a significant leucocytosis [11.95 (8.6–14.8) x 10^9^/L vs. 8.05 (6.4–9.5) x 10^9^/L, p<0.0001] with neutrophilia [6.45 (4.65–9.85) x 10^9^/L vs. 3.15 (2.3–4.1) x10^9^/L, p<0.0001], mild monocytosis[1.00 (0.7–1.23)) x10^9^/L vs. 0.6 (0.41–0.72) x10^9^/L, p<0.0001] and mild anaemia [119 ± 10.5 g/L vs. 125± 9.00 g/L, p<0.0001] in the blood count during the attack. Mean platelet volume (MPV) was higher during the attack [7.7 (7.2–8.3) fL vs. 7.5 (7.05-7.9) fL, p=0.0186]. In attack-free period we noted significant lymphocytosis[3.82 (2.8–5.27) x 10^9^/L vs. 2.75 (2.12–4.05) x 10^9^/L, p=0.0009] and eosinophilia [0.16 (0.1–0.2) x 10^9^/L vs. 0.1 (0–0.17) x 10^9^/L, p=0.0018]. The total number of platelets and basophils was not outside the reference ranges (**Table 3**).

**Table 3:** Characteristics of laboratory parameters.

	During attack Median (IQR) Avg.±SD	Attack-free period Median (IQR) Avg.±SD	p-value
CRP (mg/L, RR: 0–5)	50 (17.1–120)	0.95 (0.3–2.3)	p<0.0001
Procalcitonín (µg/L, RR:0–0.5)	0.12 (0.07–0.55)	0.06 (0.04–0.10)	p=0.0185
IgD (mg/L, RR: 7.7–132.1)	15.92 (6.76–30.68)	13.92 (6.77–25.38)	p=0.3613
SAA (mg/L, RR: 0–6.4)	132 (36.65–278.25)	4 (3.4–7.43)	p<0.0001
IL-6 (ng/L, RR: 0–7.0)	6.94 (1.87–20.64)	1.52 (1.5–2.89)	p<0.0001
Serum calprotectin (µg/mL, RR: 0–6)	3.64 (2.94–6.28)	5.92 (3.11–12.8)	p=0.036
Alfa-1 acid glycoprotein (g/L, RR: 0.4–1.2)	1.51 (1.23–1.65)	0.94 (0.74–1.32)	p=0.0013
Leucocytes (10^9^/L; RR: 3.9–10.0)	11.95 (8.6–14.8)	8.05 (6.4–9.5)	p<0.0001
Hemoglobin (g/L; RR: 120–155)	119±10.5	125±9.00	p<0.0001
Platelets (10^9^/L; RR: 140–400)	320 (255–399)	331.5 (288–404)	0.1413
MPV (fL; RR:7.5–12)	7.7 (7.2–8.3)	7.5 (7.05–7.9)	p=0.0186
Neutrophiles (10^9^/L; RR: 1.4–6.5)	6.45 (4.65–9.85)	3.15 (2.3–4.1)	p<0.0001
Lymphocytes (10^9^/L; RR: 1.2–3.4)	2.75 (2.12–4.05)	3.82 (2.8–5.27)	p=0.0009
Monocytes (10^9^/L; RR: 0.2–1.2)	1.00 (0.7–1.23)	0.6 (0.41–0.72)	p<0.0001
Eosinophils (10^9^/L; RR: 0–0.8)	0.1 (0–0.17)	0.16 (0.1–0.2)	p=0.0018
Basophils (10^9^/L; RR: 0–0.15)	0 (0–0.1)	0.03 (0–0.1)	p=0.4680

Avg., average; CRP, C-reactive protein; IgD, Immunoglobulin D; IL-6, interleukin-6; IQR, interquartile range; MPV, mean platelet volume; SAA, serum amyloid A; SD, standard deviation. Individual values ​​are given as average± standard deviation for parametric variables and as median (IR) for non-parametric variables.

### Analysis of the therapeutic approach

Conservative treatment in our PFAPA patients consisted of the episodic administration of prednisone during attacks. We usually started with a dose of prednisone of above 1mg/kg/dose as recommended by the CARRA PFAPA working group (10). In the non-responders, we increased the dose up to 2 mg/kg/dose. Out of the whole group, 122 patients used prednisone episodically during the attacks with 100% effect on ceasing the attack. Twenty patients did not have good adherence to this treatment, the most common reason was corticophobia in the patient’s parents. The average dose of prednisone in our patients was 1.2 ± 0.4 mg/kg/dose. We did not observe a significant difference between the dose needed for boys and girls (p=0.3932). As for the prophylaxis of frequent attacks,111 patients were treated with ketotifen, of which 77.5% had a positive therapeutic response. We considered a positive therapeutic response to be at least a two-fold extension of the attack-free period from the initiation of treatment to check-up (usually in the interval of 3 to 6 months from the first examination). Ketotifen treatment resulted in a significant prolongation of the attack-free period (4.4 ± 1.9 weeks vs. 14.7 ± 8.9 weeks; p<0.0001), no significant difference was noted between the genders. Ketotifen treatment (according to SPC of the product) has the only contraindication and it is hypersensitivity to ketotifen or any component of the drug. Physicians should be aware of the combination of ketotifen with CNS depressants and other antihistamines. Side effects of ketotifen treatment were noted in 4 patients (restlessness, irritability, and agitation were reported in 3 patients; in one patient probably rather side effect of antileukotriene treatment; intolerance from the gastrointestinal system in 1 patient - constipation). In one patient we ended concomitant antileukotriene treatment with improvement of the symptoms, in others we tried to lower the dose of ketotifen with partial response and parents did not want to continue in the treatment.

We also assessed the vitamin D levels and hypovitaminosis D as a risk factor for pure response to this treatment. We considered serum concentrations of vitamin D less than 30 mg/L to indicate hypovitaminosis D for this work (as used by most of the authors). We determined the serum concentration of vitamin D in 81 patients (36 girls and 42 boys). There were 42 patients with hypovitaminosis D in our group (51.9%) compared to 39 patients (48.1%) with normal vitamin D concentrations. The mean serum concentration of vitamin D in patients with hypovitaminosis was 19.95 (16.9–25.4) mg/L. In the group with hypovitaminosis D, the male gender predominated over the female (28/42 vs. 14/42; p=0.0368). In patients with hypovitaminosis D, we did not observe any statistically significant differences in the age of onset of the disease, the observed clinical and laboratory parameters, or the therapeutic response to ketotifen (despite concomitant treatment with vitamin D supplements in patients with hypovitaminosis D). All of the results are included in **Table 4**. In patients who had recurrent tonsillitis with aphthous stomatitis, we also added an oral probiotic (*Streptococcus salivarius strain K12*) to the treatment. We indicated treatment with oral probiotics in 39 patients with the clinical manifestation of aphthous stomatitis during PFAPA attacks. We recorded a positive therapeutic response (alleviation of the symptoms of aphthous stomatitis) in 71.8% of patients. Tonsillectomy (TE) was chosen in patients who had persistent frequent attacks lasting for more than a year– altogether, 29 patients (15 boys and 14 girls) for TE. The average age of TE was 5.7 ± 1.7 years (boys 5.6 ± 1.3 years, girls 5.8 ± 2 years; p=0.7833) and 96.6% of patients (28/29 patients) benefited from TE. Complete resolution of symptoms was reached in 4.8 ± 1.2 months after TE. Only 1 patient has not yet experienced the complete resolution of symptoms after TE (approximately 1 year since TE). In this patient, we observed just minor symptoms, even after tonsillectomy. All data are included in **Table 5**.

**Table 4:** Vitamin D and PFAPA syndrome.

	Hypovitaminosis D N=42	Without hypovitaminosis D N=39	p
Serum concentration of vitamin D (mg/L)	19.95 (16.9-25.4)	37.6 (32.7-47.2)	p<0.0001
Fever (N, %)	42 (100 %)	39 (100 %)	n.s.
Pharyngitis (N, %)	40 (95.2 %)	35 (89.7 %)	0.3454
Aphthous stomatitis (N, %)	21 (50 %)	18 (46.2 %)	0.7292
Cervical lymphadenopathy (N, %)	37 (64.3 %)	33 (84.6 %)	0.6478
CRP attack (mg/L, N: 0-5)	55.9 (15.63-143.65)	50 (16.88-109.1)	0.4560
CRP attack-free(mg/L, N: 0-5)	0.8 (0.3-1.75)	0.8 (0.3-2.85)	0.9709
IL-6 attack (ng/L, N: 0-7)	6.4 (2.05-9.14)	4.97 (1.57-41.42)	0.7470
IL-6 attack-free (ng/L, N: 0-7)	1.72 (1.5-2.73)	1.5 (1.5- 2.08)	0.2940
Positive effect of ketotifen (N, %)	23/34 (67.6 %)	22/ 27 (81.5 %)	0.0912
Attack-free interval before ketotifen treatment (weeks)	4 (3-5)	4 (3-5)	0.8046
Attack-free interval after ketotifen treatment (weeks)	16 (10.5-23.5)	12 (8-17)	0.1065

CRP, C-reactive protein; IL-6, interleukin-6; N, number of patients.

**Table 5:** Therapeutic approach in our PFAPA patients.

	Whole group		Boys		Girls		p-value
	N=142		N=71		N=71		
Prednisone episodically (N, %)	122 (85.9%)		57 (79.2%)		65 (90.3%)		
Positive prednisone test (N, %)	122 (100%)		57 (100%)		65 (100%)		
Dose of prednisone (mg/kg/dose)	1.2±0.4		1.26±0.38		1.2±0.32		0.3932
Ketotifen (N, %)	111 (78.2%)		56 (77.8%)		55 (76.4%)		
Positive effect of ketotifen (N, %)	86 (77.5%)		42 (75%)		44 (80%)		0.5283
Dose of ketotifen (mg/kg/day)	0.08±0.01		0.09±0.02		0.086±0.017		0.2664
Side effects of ketotifen (N, %)	4 (3.6%)		0		4 (7.3%)		0.2997
Attack-free interval before ketotifen treatment (weeks)	4.4±1.9	p*<0.0001	4.5±1.9	p*<0.0001	4.3±1.9	p*<0.0001	0.2252
Attack-free interval after ketotifen treatment (weeks)	14.7±8.9		14.9±8.9		14.5±8.9		0.9082
Oral probiotic *(Streptococcus salivarius K12*) (N, %)	39 (27.5%)		18 (25%)		21 (29.2%)		
Positive effect of oral probiotic (N, %)	28 (71.8%)		14 (77.8%)		14 (66.7%)		0.442
Tonsillectomy (N, %)	29 (20.4%)		15 (20.8%)		14 (19.4%)		0.8354
Age of TE (years)	5.7±1.7		5.6±1.3		5.8±2		0.7833
Positive effect of TE (N, %)	28 (96.6%)		14 (93.3%)		14 (100%)		
Symptom resolution after TE (years)	0.4±0.1		0.3±0.2		0.5±0.3		0.9339

N, number of patients; TE, tonsillectomy. p-value is counted between the genders, p*- value is counted within the groups for the attack-free interval before and after ketotifen treatment.

## Discussion

PFAPA was reported to be a sporadic disease. In recent years, however, there have been increasing numbers of studies that have recorded a positive family history in first-degree relatives of patients with PFAPA. It was first documented by case report series (29–31). Cochard et al. reported the occurrence of recurrent fever in 45% of first-degree relatives of patients with PFAPA in a study of 84 patients (32). However, the limitation of this study was the fact that the authors focused only on the occurrence of recurrent fever in the questionnaire. For this reason, such a high percentage of patients with a positive family history cannot be considered relevant. A more detailed observation was made by Manthiram et al. (18). In their study, they not only focused on the occurrence of periodic fever in the patient’s family, but also on other clinical symptoms of PFAPA (recurrent aphthous stomatitis, recurrent tonsillitis). The study gathered together 80 patients with PFAPA syndrome and a complementary control group. The results of the study showed that 23% of patients with PFAPA had ≥1 first-degree relative (parent, sibling) with clinical symptomatology meeting the criteria for PFAPA. Parents of individuals with PFAPA syndrome had a significantly higher incidence of recurrent pharyngitis and recurrent aphthous stomatitis compared to parents of the control group. The authors demonstrated that PFAPA probably has a genetic predisposition. The presence of gene variants with low penetrance in first-degree relatives is assumed (explaining incomplete PFAPA phenotype in relatives). Takeuchi et al. (33) found positive family history for recurrent fever, tonsillitis or tonsillectomy in 62% patients (out of a total of 257 patients) with PFAPA. They therefore suggested that PFAPA could be a disease with an autosomal dominant mode of inheritance with the involvement of a gene with low penetrance. Inclusion of a positive family history among the secondary diagnostic criteria for PFAPA should be considered. In our work we recorded a positive family history in 31.6% of patients. Our findings are almost consistent with those of Hofer et al. for the European population (multicentre study of the European cohort of patients). They identified a positive family history (recurrent tonsillitis or recurrent fever) in 26.9% of patients with PFAPA (5). Therefore, PFAPA syndrome is not a sporadic disease and shows signs of incomplete penetration in about one third of patients. Our study also confirms this hypothesis. We included a detailed analysis of clinical and selected laboratory parameters and observed fever in 100%, pharyngitis in 94.4%, aphthous stomatitis in 54.9% and cervical lymphadenopathy in 91.6% of patients. From other not such so specific symptoms of PFAPA: arthralgias were present in 19.7%, abdominal pain in 29.6%,skin rash in 7.8% and fatigue in 16.2% of patients. Our findings are similar to other literature data (comparison of our results with similar studies is included in **Table 6**). When comparing individual symptoms, the high percentage of patients with abdominal pain in the study by Perko et al. is interesting, because abdominal pain is a sensitive symptom for familial Mediterranean fever. This was a study from Slovenia, where the incidence of monogenic periodic fever syndromes is supposed to be higher compared to the Slovak population. In these patients, a genetic examination was carried out for monogenic periodic fever syndromes and the occurrence of non-pathogenic variants in *MEFV* and *MVK* genes were confirmed (11). In our work, we recorded one patient with genital ulcers as a possible symptom of PFAPA, which is not a typical manifestation of PFAPA. In the literature, there are rare case reports of patients with similar clinical symptomatology (35, 36). In the laboratory parameters, we observed a significant elevation of CRP as well as an elevation of other pro-inflammatory cytokines like serum amyloid A, interleukin-6 and alpha-1 acid glycoprotein during the attacks compared to the attack-free periods, and also a slight elevation of procalcitonin (possible coincidence of PFAPA attack and bacterial superinfection) in some patients. We did not notice a difference in the serum concentration of IgD during and in attack-free periods. We detected a significant elevation of serum calprotectin as a marker of subclinical inflammation in attack-free periods. In the blood count, we found significant leucocytosis with neutrophilia, mild monocytosis and mild anaemia during attacks. Mild anaemia could be attributed to attacks of inflammation, in which the well-known phenomenon is the activation of innate immunity and proinflammatory cytokines. Activation of cytokines (especially IL-1 and IL-6) can lead to shifts in iron utilisation (elevation of levels of serum ferritin and depression of levels of serum transferrin) and an inflammation-mediated reduction in red blood cells. Something similar can be seen in anaemia of chronic diseases. The value of the mean platelet volume was higher during the attack, which is not consistent with previously published data. Some authors observed a low mean platelet volume in patients with PFAPA during the attack and considered it a typical laboratory picture of PFAPA syndrome. A decreased number of eosinophils during a PFAPA attack was not noted in our study, but we noted significant eosinophilia and lymphocytosis in attack-free periods.

**Table 6:** Clinical symptoms of PFAPA (comparison of studies).

Author Citation number N=number of patients	Our studied cohort N=142	Perko et al. (11) N=81	Król et al. (34) N=125	Feder et al. (7) N=105
Age of first symptoms	2.31±2.02 years	2.1±1.5 years	23 months	39.6 months
Attack duration (days)	2.8±1.2	4.2	3.5	4.1
Attack-free interval (weeks)	4	4	4	4
Fever	100%	100%	100%	100%
Pharyngitis	94.4%	98%	91%	85%
Aphthous stomatitis	54.9%	56%	41%	38%
Genital ulcers	0.7%	0%	0%	0%
Cervical lymphadenopathy	91.6%	94%	78%	62%
Arthralgias	19.7%	31%	10%	–
Abdominal pain	29.6%	51%	23%	41%
Skin rash	7.8%	12%	3%	–
Fatigue	16.2%	–	–	prodrome in 62%

According to the available data, a single dose of prednisone (1–2 mg/kg) or betamethasone (0.1–0.2 mg/kg) administered at the beginning of an attack leads to a rapid termination of the attack within a few hours (6, 7, 19, 37). EUROFEVER registry data confirm that up to 90% of patients with PFAPA have a positive therapeutic response to oral corticosteroids (37). At the same time, it can be used as an important diagnostic-therapeutic test, which can confirm this disease and can be helpful in distinguishing PFAPA from other monogenic periodic fever syndromes. If the first dose of corticosteroids is not effective, most authors recommend an extended schema - administration of the second dose on the next day. Even in our clinical practice, we found that about 25% of patients required a longer scheme of corticosteroids administration (usually 3–4 days). Hofer et al. observed a rapid termination of the attack after one dose of corticosteroids in 63% of patients, 32% of patients responded partially and required another dose on the second day and 8% did not respond to the administration of corticosteroids (5).Wurster et al. published a cohort of 60 PFAPA patients, reporting corticosteroid efficacy in 84% of patients (38). Tasher et al. reported that prednisone at a single dose of 0.6 mg/kg/dose was effective in terminating an attack within the first 10 hours after onset in 94% of patients (4). A recent study by Yazgan et al. proved that there was no difference in effectiveness using a single dose of prednisone 2 mg/kg/dose vs. 0.5 mg/kg/dose (20). The use of corticosteroids in PFAPA syndrome is limited by the fact that the intervals between attacks can shorten at the beginning of their usage in 25–50% of patients (19, 28). This fact and corticophobia can lead to poor compliance with treatment. Therefore, it is necessary to inform the patient about this fact before and to communicate the safety of such treatment approach. Among the side effects of corticosteroid treatment, restlessness and insomnia were noted in some patients, but systemic side effects were not observed. In our cohort of patients, 122 patients (85.9%) used prednisone episodically during an attack with a 100% effect in ceasing the attack. 20 patients did not have good compliance with this treatment. The most common reason was corticophobia despite an adequate explanation of this treatment to their parents. The mean effective dose of prednisone for in our patients was 1.2 ± 0.4 mg/kg/dose, and we did not observe a significant difference in the dose needed for boys and girls. About 25% of patients required a repeated administration of corticosteroids to suppress the attack. Our findings are consistent with previously published studies. A new contribution of our study is the confirmation of the positive therapeutic effect of ketotifen in the prophylaxis of frequent PFAPA attacks. We were inspired by case series published by Toplak et al. The rationale for such approach can be the fact that ketotifen reduces the production of IL-1β and some chemokines and modulates the T-cell response (26), which may have a positive effect on the immune dysregulation observed in PFAPA. Other studies investigating the effect of ketotifen also confirmed that ketotifen reduces the production or suppresses the secretion of the main pro-inflammatory cytokines, namely IL-1β, IL-6 and TNF-α (39–42). Based on our experience and the data achieved, we recommend the administration of ketotifen if the episodes have a high frequency (every 10–14 days) and are long-lasting (more than 6 months). Treatment with ketotifen was indicated in 111 patients, of which 77.5% had a positive therapeutic response. We considered a positive therapeutic response to be at least a two-fold extension of the attack-free period from the initiation of treatment to the first check-up (usually in the interval of 3–6 months from the first examination). Ketotifen treatment resulted in a significant prolongation of the attack-free period (4.4 ± 1.9 weeks vs. 14.7 ± 8.9 weeks; p<0.0001), no significant difference was noted between the genders. An average dose of ketotifen was 0.08 ± 0.01 mg/kg/day. Side effects of ketotifen were noted in 4 patients (restlessness, irritability, agitation reported in 3 patients; intolerance from the gastrointestinal system in 1 patient- constipation). So far, there have been no published studies with such a large cohort of PFAPA patients focused on the positive prophylactic effect of ketotifen in PFAPA. However, we are aware, that our study had some limitations. The first is the absence of a matched cohort in which ketotifen was not administered. As you could notice, 31 patients did not receive the treatment, because either they were non-compliant with the regular treatment or the disease course was different with low frequency of attacks. Logically, they were not good candidates for the control group (low frequency of flares and non-compliance). Also our study was missing laboratory marker to prove the efficacy of ketotifen. In PFAPA we do not have one inflammatory marker which would correlate with disease activity (e.g., as it is with FMF and SAA). We tried to measure serum calprotectin and SAA (as the markers of subclinical inflammation), but they did not correlate well with disease activity in PFAPA. We also performed the analysis of concomitant allergic diseases (treatment of them could affect the results). From the whole group of patients (n=142) we found that 42.9% had allergic rhinitis; 8.5% asthma; 18.3% atopic dermatitis. In our outpatient department we do not prefer dual antihistamine therapy, so all the patients with the need of antihistamine therapy were treated with ketotifen alone. From nasal corticoids we used mometasone furoate, which has a very low total systemic bioavailability (it is reported to be <0.5%). In therapy of asthma, we usually start with fluticasone propionate in low doses (systemic bioavailability reported of approximately 17%), antileukotrien was used in 3 patients. We did not find different results between patients treated with this drug for allergic conditions and patients taking ketotifen alone. Another confounding factor in our study could be concomitant supplementation with vitamin D in PFAPA patients with hypovitaminosis D. In previous studies investigating the relationship between vitamin D and PFAPA syndrome hypovitaminosis D was considered to be a risk factor for more frequent attacks of PFAPA syndrome (43, 44). Vitamin D supplementation in patients with PFAPA syndrome led to a significant reduction in the number of episodes and modification of their course (shortening the duration of the episode and the number of episodes per year) (44). The last study on vitamin D and PFAPA syndrome declared that in the group of patients with PFAPA syndrome with normal vitamin D concentration (i.e., more than 30 ng/ml), the duration of the PFAPA episode was shorter and the interval between attacks was longer than in the group with hypovitaminosis D (45). According to our statistical analysis, we did not observe any statistically significant differences in clinical and inflammatory parameters between PFAPA patients with or without hypovitaminosis D. Hypovitaminosis D did not seem to affect the effect of ketotifen treatment. Both groups had similar results when looking at the prolongation of attack-free periods. We would like to emphasise at this point, that this is the pilot study and further research would be needed to prove our findings. As for the surgical treatment, we indicated TE in 29 patients (15 boys and 14 girls). The mean age of TE in our cohort was 5.7 ± 1.7 years. 96.6% of patients benefited from TE and symptoms subsided after 0.4 ± 0.1 year from TE. Only 1 patient did not experience complete regression of symptoms yet (approx. 1 year after tonsillectomy). In the group of patients who were indicated for TE, we noted a significantly higher serum concentration of CRP during the attack (100 (43.9–136.07) mg/l vs. 35.4 (15.6–109.8); p=0,00286). Compared to the available studies, the effectiveness of TE in our patients is relatively high. Garavello et al. published the study with 39 patients, of which 19 underwent adenotonsillectomy and 20 were treated conservatively (46). The effect of tonsillectomy was noted in 63% of patients within 6 months after surgery; 100% of patients had no PFAPA attack after 18 months from surgery. Only one patient treated conservatively had a complete remission of PFAPA syndrome. In a prospective work by Licameli et al., 102 patients underwent adenotonsillectomy; in 97% of patients the symptoms resolved immediately after the operation, 1 patient achieved remission 6 months after the procedure and fever persisted in 1 patient 43 months after the operation. The last patient from this cohort underwent genetic testing and was reclassified as a patient with mevalonate kinase deficiency (28). Our findings are most consistent with those of Licameli et al. We attribute such large differences in the observed findings from mentioned studies to the use of incorrect diagnostic criteria for PFAPA, the incorrect indication of tonsillectomy in these patients, and different surgical approaches (adenotonsillectomy vs. tonsillectomy). However, it has not been proven that the combination of adenoidectomy with tonsillectomy versus tonsillectomy alone would have a greater therapeutic effect in PFAPA (47)].

## Conclusion

PFAPA is the disease of unknown aetiology. With our work, we tried to provide a comprehensive view of PFAPA, its clinical and laboratory picture and therapeutic possibilities. We characterised the Slovak cohort of PFAPA patients for the first time. However, our results are also relevant for patients in other countries. It is the second work to date which has proven a possible prophylactic effect of ketotifen on frequent PFAPA attacks and the first work with such a large cohort of patients treated with ketotifen. Further research in this area is needed to confirm our findings.

## Data availability statement

The raw data supporting the conclusions of this article will be made available by the authors, without undue reservation.

## Ethics statement

The studies involving humans were approved by Ethics Committee of Jessenius Faculty of Medicine in Martin, Slovakia. The studies were conducted in accordance with the local legislation and institutional requirements. Written informed consent for participation in this study was provided by the participants’ legal guardians/next of kin.

## Author contributions

LK: Conceptualization, Data curation, Investigation, Methodology, Validation, Writing – original draft, Writing – review & editing, Formal analysis, Resources, Software. PB: Conceptualization, Formal analysis, Methodology, Writing – review & editing, Validation. AB: Formal analysis, Methodology, Validation, Writing – review & editing. EJ: Data curation, Formal analysis, Investigation, Methodology, Software, Writing – review & editing. DK: Data curation, Investigation, Project administration, Writing – review & editing. OP: Conceptualization, Data curation, Investigation, Project administration, Validation, Writing – review & editing. BS: Data curation, Investigation, Project administration, Writing – review & editing. AM: Data curation, Formal analysis, Investigation, Supervision, Writing – original draft, Writing – review & editing. OF: Conceptualization, Data curation, Investigation, Writing – review & editing. KV: Formal analysis, Methodology, Validation, Writing – original draft, Writing – review & editing. MJ: Conceptualization, Data curation, Investigation, Methodology, Supervision, Validation, Writing – original draft, Writing – review & editing.
